# A *de novo* heterozygous *POU3F3* genotype for the p.(Q214*) variant in a fetus with transient isolated bilateral mild ventriculomegaly: a case report and review of the literature

**DOI:** 10.3389/fped.2023.1177137

**Published:** 2023-08-01

**Authors:** Hongyun Zhang, Siyuan Linpeng, Yanling Teng, Can Peng, Desheng Liang, Zhuo Li, Lingqian Wu

**Affiliations:** ^1^ Center for Medical Genetics, Hunan Key Laboratory of Medical Genetics, School of Life Sciences, Central South University, Changsha, China; ^2^ Department of Genetics and Eugenics, Hunan Provincial Key Laboratory of Regional Hereditary Birth Defects Prevention and Control, Changsha Hospital for Maternal and Child Health Care Affiliated to Hunan Normal University, Changsha, China; ^3^ Laboratory of Molecular Genetics, Hunan Jiahui Genetics Hospital, Changsha, China

**Keywords:** transient isolated bilateral mild ventriculomegaly, *POU3F3*, whole-exome sequencing, snijders blok-Fisher syndrome, truncating variant

## Abstract

The prenatal prevalence of isolated ventriculomegaly is 0.039%–0.087%. Most isolated mild ventriculomegaly (MV) fetuses (>90%) have a favorable prognosis. However, 5.6% to 7.9% of fetuses with isolated MV have adverse neurodevelopmental outcomes. In this study, we reported the first case of prenatal Snijders Blok-Fisher syndrome (OMIM: #618604) caused by a truncating variant of *POU3F3* (OMIM: *602480) in a fetus with transient isolated bilateral MV. The results of karyotype analysis, chromosomal microarray analysis, and TORCH infection evaluation for the fetus were all negative. However, a *de novo* likely pathogenic nonsense variant of NM_006236.3 (*POU3F3*): c.640C > T [rs1254251078] p.(Q214*) was identified by whole-exome sequencing (WES). Despite sufficient genetic counseling, the mother refused to undertake further brain magnetic resonance imaging (MRI) and decided to keep the fetus. She gave birth to a male infant through a full-term vaginal delivery. With a long-term follow-up, the infant unfortunately gradually presented with delayed motor development. The postnatal brain MRI of the proband showed dysplasia of the corpus callosum and ventriculomegaly. Considering the high probability of misdiagnosis for such cases, we further summarized the prenatal phenotypes from 19 reported patients with variants in *POU3F3.* The results revealed that 14 patients displayed a normal prenatal ultrasonographic manifestation, while only approximately 26.32% of fetuses showed MV or cysts without structural deformity. Thus our findings expand the variant spectrum of *POU3F3* and suggest the importance of undertaking WES and brain MRI when the fetus has isolated bilateral MV.

## 1. Introduction

Ventriculomegaly is one of the most common findings in prenatal ultrasounds, with a prevalence of 0.3–1.5 per 1,000 live births (1). Isolated ventriculomegaly is considered when no additional sonographic or chromosomal aberrations are identified, with a prenatal prevalence of 0.039%–0.087% (2, 3). When the diameter of the atrium of the lateral ventricles reaches 10–15 mm, it can be classified as mild lateral ventriculomegaly (MV), with a prenatal prevalence of 0.07% (3, 4). Most isolated mild ventriculomegaly (MV) fetuses (>90%) have a favorable prognosis. In contrast, an adverse neurodevelopmental outcome in fetal isolated MV can account for 5.6% to 7.9% of cases (3). Hence, for those fetuses with isolated MV, continuous ultrasound monitoring, brain magnetic resonance imaging (MRI), TORCH infection evaluation, and genetic testing (2) are suggested to explore the potential etiology to facilitate pregnancy management and predict neurodevelopmental outcomes.

In 2019, Petrovski et al. identified a *POU3F3* heterozygous missense variant in a fetus with ventriculomegaly (5). POU3F3 (POU domain, class 3, transcription factor 3) is a member of the class III POU family of transcription factors, which is essential to cortical neuronal migration and neurogenesis (6). The variants in *POU3F3* (OMIM: *602480) can cause an autosomal dominant neurodevelopmental disorder named Snijders Blok-Fisher syndrome (SBS). SBS (OMIM: #618604) is mainly manifested by global developmental delay and intellectual disability. A few patients may have epilepsy. The human gene mutation database (HGMD) has recorded 23 variants of *POU3F3* up to February 2023. However, the truncating variants in *POU3F3* have not been associated with ventriculomegaly.

This report described a fetus with a truncating *de novo* likely pathogenic variant of *POU3F3* verified by trio whole-exome sequencing (WES) with transient isolated bilateral MV. The fetal results of karyotype analysis, chromosomal microarray analysis (CMA), and TORCH infection evaluation were negative. For the first time, this report associates a truncating variant in *POU3F3* with the transient isolated bilateral MV.

## 2. Case report

**Figure 1:**
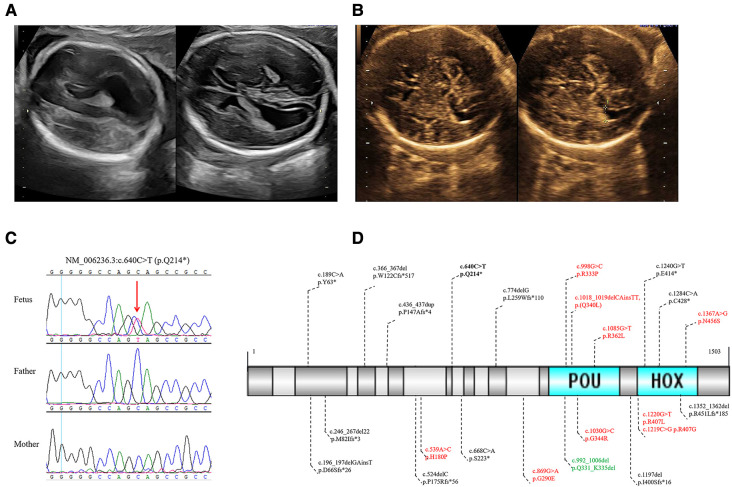
(**A**) The 24 weeks ultrasound of the fetus showed bilateral MV. (**B**) The 32 weeks ultrasound of the fetus showed a normal high value of bilateral ventricles. (**C**) Sanger sequencing validated the novel *de novo* likely pathogenic variant of *POU3F3* in the fetus and healthy parents. (**D**) In all, 23 *POU3F3* variants were recorded in HGMD up to February 2023. One variant was the gross deletion of the entire *POU3F3*, which is not in (**D**). Black, truncating variants; bold black, the variant in this study; red, missense variants; green, inframe deletion.

In the present study, we identified a novel *de novo POU3F3* nonsense variant in a Chinese fetus with isolated bilateral MV. The mother and father were 27 and 31 years old, respectively. There was no history of exposure to toxic substances and radiation during preconception planning and pregnancy. Regular maternity checkups were undertaken without abnormalities. There was no abnormal abdominal pain or vaginal bleeding during pregnancy. The results of non-invasive prenatal testing, nuchal translucency, and TORCH infection evaluation were negative. Amniocentesis was performed due to bilateral MV (left: 11.3 mm, and right: 10.9 mm), and a right choroid plexus cyst in the fetus was found by ultrasound at 24 weeks of gestation age (Figure 1A).

The amniotic fluid cells were collected to undertake the karyotype analysis, CMA, and WES, of which WES was performed in the fetus and healthy parents. The results of karyotyping and CMA were negative. The average depth in WES was 107X. The variants were analyzed in inheritance patterns, genotype-phenotype associations, and the pathogenicity evaluation after paired-end sequencing, alignment, variant calling, and annotation. We identified a novel *de novo* nonsense variant of NM_006236.3 (*POU3F3*): c.640C > T [rs1254251078] p.(Q214*) through trio WES (Figures 1C, Supplementary Figure S1). This variant was neither found in 1000G nor GnomAD; the CADD score was 36. Seven algorithms predicted that the variant was harmful (Supplementary Table S1). The final pathogenicity of c.640C > T was a likely pathogenic variant (PVS1_strong, PS2_moderate, and PM2_supporting) according to the American College of Medical Genetics and Genomics (ACMG) guidelines. The Sanger sequencing verified the likely pathogenic variant found by WES (Figure 1C). After consultation, the laboratory technician, genetic consultant, and clinician suggested that the parents undertake a brain MRI and pregnancy management.

During the follow-up, fetal ultrasound (32 weeks) showed a normal high value of bilateral ventricles (left: 9.5 mm, and right: 9.8 mm) and a right choroid plexus cyst (Figure 1B). The mother refused to undertake a brain MRI and gave birth to a full-term vaginal delivery of a male infant. No abnormality at birth and delivery was noted, and neonatal screening was negative. At the last visit, the child was two years old. He could sit at seven months old and walk with support after rehabilitation training at 1.5 years old. He had facial dysmorphism (ocular hypertelorism, protruding ears, and esotropia) and delayed fine and gross motor function. Moreover, the proband had mild developmental delays. However, he did not have speech delay, epilepsy, sleep disorder, autism, attention deficit hyperactivity disorder, joint hypermobility, and scoliosis. Postnatal brain MRI of the proband showed dysplasia of the corpus callosum and ventriculomegaly. Unfortunately, his parents were not willing to provide his brain MRI images.

We summarized the phenotypes from 48 patients with variants in *POU3F3* (Table 1). In all, 44 (91.67%) patients had developmental delays and/or intellectual disabilities, and the clinical information of the other four patients was unknown. There was a phenotype difference between the non-truncating and truncating variants. For example, epilepsy was more common in patients with a non-truncating variant of *POU3F*3 (4/17, 23.53%), while only one patient had epilepsy in 27 patients with a truncating variant of *POU3F*3 (1/27, 3.70%). Moreover, 14 of 19 fetuses with variants in *POU3F3* had a normal prenatal ultrasound during pregnancy. Besides, abnormal prenatal ultrasound of fetuses (5/19, 26.32%) mostly showed MV or cysts without structural deformity (5–7). In contrast, 10 of 22 (45.45%) patients had an abnormal postnatal brain MRI.

**Table 1:** Summary of phenotypes in 48 individuals with *POU3F3* variants.

	Non-truncating variants (5–11)	Truncating variants (6, 11)	All variants (percentage)
Patients	20	28	48
Age at last visit (years)	0.8–39^a^	1.08–41	0.8–41
Male: Female	8:9	18:10	26:19
Developmental delay and/or intellectual disability	16 (36.36%^a^)	28 (63.64%)	44 (91.67%)
① Borderline or mild	7 (43.75%)	11 (39.29%)	18 (40.91%)
② Moderate	3 (18.75%)	8 (28.57%)	11 (25.00%)
③ Severe	3 (18.75%)	1 (3.57%)	4 (9.09%)
④ Profound	1 (6.25%)	0 (0.00%)	1 (2.27%)
⑤ Severity unknown	2 (12.50%)	8 (28.57%)	10 (22.73%)
Motor (yes or no)			
Motor delay	12 (60.00%)	24 (85.72%)	36 (75.00%)
No motor delay	2 (10.00%)	2 (7.14%)	4 (8.33%)
Motor delay (unknown)	6 (30.00%)	2 (7.14%)	8 (16.67%)
Speech (yes or no)			
Speech delay or disorder	16 (80.00%)	26 (92.86%)	42 (87.50%)
No speech delay or disorder	0 (0.00%)	1 (3.57%)	1 (2.08%)
Speech delay or disorder (unknown)	4 (20.00%)	1 (3.57%)	5 (10.42%)
Sleep (yes or no)			
Sleep disorder	5 (25.00%)	9 (32.14%)	14 (29.17%)
No Sleep disorder	7 (35.00%)	13 (46.43%)	20 (41.66%)
Sleep disorder (unknown)	8 (40.00%)	6 (21.43%)	14 (29.17%)
Epilepsy (yes or no)			
No epilepsy	13 (65.00%)	26^b^ (92.86%)	38 (79.17%)
Epilepsy	4 (20.00%)	1 (3.57%)	6 (12.50%)
Epilepsy (unknown)	3 (15.00%)	1 (3.57%)	4 (8.33%)
Behavior (yes or no)			
① Autism or autistic features	6 (30.00%)	7 (25.00%)	13 (27.08%)
② No autism or autistic features	6 (30.00%)	20 (71.43%)	26 (54.17%)
③ Autism or autistic features (unknown)	8 (40.00%)	1 (3.57%)	9 (18.75%)
④ No Attention deficit hyperactivity disorder	11 (55.00%)	25 (89.29%)	36 (75.00%)
⑤ Attention deficit hyperactivity disorder	3 (15.00%)	3 (10.71%)	6 (12.50%)
⑥ Attention deficit hyperactivity disorder (unknown)	6 (30.00%)	0 (0.00%)	6 (12.50%)
Skeletal system (yes or no)			
① No joint hypermobility	12 (60.00%)	18 (64.29%)	30 (62.50%)
② Joint hypermobility	4 (20.00%)	8 (28.57%)	12 (25.00%)
③ Joint hypermobility (unknown)	4 (20.00%)	2 (7.14%)	6 (12.50%)
④ No scoliosis	14 (70.00%)	26 (92.86%)	40 (83.34%)
⑤ Scoliosis	2 (10.00%)	2 (7.14%)	4 (8.33%)
⑥ Scoliosis (unknown)	4 (20.00%)	0 (0.00%)	4 (8.33%)
Prenatal brain ultrasound (yes or no)			
① Abnormalities reported on Ultrasound	3 (15.00%)	2 (7.14%)	5 (10.42%)
Dilation of ventricles	1	1	2
Brain cysts	1	1	2
Hydrocephalus	0	1	1
Polyhydramnios	1	0	1
≥2 indications	0	1	1
② Ultrasound: normal	4 (20.00%)	10 (35.71%)	14 (29.17%)
③ Ultrasound: unknown	13 (65.00%)	16 (57.15%)	29 (60.41%)
Postnatal brain magnetic resonance imaging (yes or no)			
① Abnormalities reported on brain MRI	4 (20.00%)	6 (21.43%)	10 (20.83%)
Cerebral atrophy and/or dilation of ventricles	4	2	6
Corpus callosum abnormality	1	4	5
Delayed myelinization	0	2	2
Hypoplasia of olfactory bulbs	0	1	1
Multifocal hydromyelia involving cervical and thoracic spinal cord	0	1	1
≥2 indications	1	3	5
② Brain MRI: normal	4 (20.00%)	8 (28.57%)	12 (25.00%)
③ Brain MRI: unknown	12 (60.00%)	14 (50.00%)	26 (54.17%)

^a^ The results of developmental delay and/or intellectual disability in 4 out of 20 patients were unknown. The data of those four patients were taken from previous studies (5, 9, 10, 12).

^b^ One out of 26 patients showed spikes in an electroencephalogram without a seizure.

## 3. Discussion

Up to February 2023, the HGMD had recorded 23 variants in *POU3F3*, of which 54.55% were truncating variants (Figure 1D). The penetrance of reported microdeletions or duplications in *POU3F3* is of high penetrance (12). The post-transcriptional monitoring mechanism of nonsense-mediated mRNA decay (NMD) helps impede the translation of abnormally truncated proteins (13). However, *POU3F3* is an intronless gene, which may lead to NMD escape and the impossibility to apply the PVS1 criterion of the ACMG/AMP pathogenicity classification. c.196_197delinsT (p.D66Sfs*26), c.668C > A (p.S223*), and c.1197delG (p.I400Sfs*16) were around c.640C > T (p.Q214*) in *POU3F3* (Figure 1D). Snijders Blok et al. found that NMD escape was found in HEK293 cells with c.196_197delinsT (p.D66Sfs*26), c.668C > A (p.S223*), or c.1197delG (p.I400Sfs*16). The pathogenic mechanisms of c.196_197delinsT, c.668C > A, and c.1197delG were aberrant cytoplasmic expression and the decrease of dimerization capacity (6), which indicated the truncated regions in c.196_197delinsT, c.640C > T, c.668C > A, and c.1197delG were critical to the function of POU3F3. Besides, the pathogenic mechanism of POU3F3 was loss-of-function, which made it possible to apply the PVS1_strong criterion in the pathogenicity evaluation of c.640C > T, according to ACMG guidelines (14).

There is a difference in the proportion of prenatal (5/19, 26.32%) and postnatal (10/22, 45.45%) brain abnormalities among patients with variants in *POU3F3*. Two possible reasons may cause this phenomenon. First, ultrasounds have a lower resolution in detecting brain tissue than MRIs, which can lead to fine structural deformities not being diagnosed by ultrasound (15, 16). Second, the patients with variants in *POU3F3* may not have brain structural deformity at birth, and the postnatal brain structural deformity may be progressive.

Most isolated MV fetuses (>90%) have a favorable prognosis. However, 5.6% to 7.9% of fetuses with isolated MV have adverse neurodevelopmental outcomes (3). Therefore, it is essential to undertake continuous ultrasound monitoring, genetic testing, brain MRIs, and TORCH infection evaluation for a comprehensive evaluation of fetal MV under the current guideline (2, 17). Some studies have found that MV has recently been associated with monogenic disorders (18, 19). For example, MV is found to be the most common prenatal ultrasound phenotype in MDS/PAFAH1B1-related lissencephaly (18). Moreover, the diagnostic yield of fetal isolated MV is 13% (3/23) through WES (20). According to the guideline of fetal MV from the Society for Maternal-Fetal Medicine in 2018 (17), these fetuses with isolated MV caused by monogenic disorders would have been missed diagnoses. Therefore, we recommended that WES should be the last diagnostic option for fetuses with isolated bilateral MV but a normal CMA result. Exploring the genetic etiology of isolated MV facilitates pregnancy management and predicts neurodevelopmental outcomes, and applying WES and brain MRI promotes fetal intrauterine phenotype-genotype association enrichment.

In conclusion, we report the first case of prenatal Snijders-Blok-Fisher syndrome caused by a *de novo* heterozygous truncating variant of *POU3F3* in a fetus with transient isolated bilateral MV. It suggests the importance of undertaking WES and brain MRI when the fetus has isolated bilateral MV. Exploring the genetic etiology of isolated MV facilitates pregnancy management and predicts neurodevelopmental outcomes. Besides, our findings expand the variant spectrum and fetal intrauterine phenotype-genotype association of *POU3F3*.

## Data Availability

The data presented in the study are deposited in the Genome Sequence Archive (GSA) (https://ngdc.cncb.ac.cn/gsa/) BioProject: PRJCA018171, Accession number HRA005034. Further requests can be directed to the corresponding author.
