# Supplementary material for: A de novo heterozygous POU3F3 genotype for the p.(Q214*) variant in a fetus with transient isolated bilateral mild ventriculomegaly: a case report and review of the literature
Source: Front Pediatr. 2023 Aug 1;11:1177137. doi: 10.3389/fped.2023.1177137 (PMC10427865; doi:10.3389/fped.2023.1177137)
Supplement: Supplementary file 1 [file Datasheet1.docx]

Supplementary Material

**Supplementary Table S1 The predicted results of c.640C>T in *POU3F3***

| Algorithm | Score | Results of prediction |
| --- | --- | --- |
| MutationTaster | 1.000 | Disease_causing |
| CADD | 36 | Damaging |
| DANN | 0.997 | Damaging |
| FATHMM_MKL | 0.524 | Damaging |
| Eigen | 0.520 | Damaging |
| GenoCanyon | 1.000 | Damaging |
| GERP++ | 2.81 | Conserved |


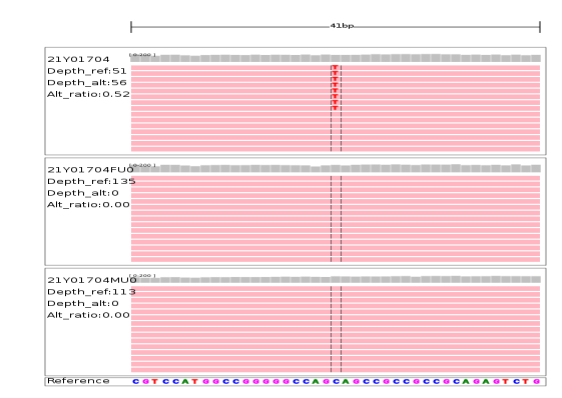


**Supplementary Figure 1.** The screenshot of c.640C>T of *POU3F3* in the bam file from WES in the fetus and healthy parents. The average depth in WES was 107X. 21Y01704: fetus, 21Y01704FU0: father, 21Y01704MU0: mother.
